# Genome-Wide Identification of *PIN* and *PILS* Gene Families in *Areca catechu* and the Potential Role of *AcPIN6* in Lateral Brace Root Formation

**DOI:** 10.3390/plants12010033

**Published:** 2022-12-21

**Authors:** Yicheng Wang, Guangzhen Zhou, Haifen Luo, Xinyu Li, Kelan Zhang, Yinglang Wan

**Affiliations:** Hainan Key Laboratory for Sustainable Utilization of Tropical Bioresources, College of Tropical Crops, Hainan University, Haikou 570228, China

**Keywords:** *Areca catechu*, PIN-FORMED, PILS, brace root

## Abstract

PIN-FORMED (PIN) and PIN-LIKES (PILS) are two families of auxin transporters that control the directional cell-to-cell transport and intracellular accumulation of auxin, thereby influencing plant growth and development. Most knowledge of PINs and PILSs was obtained from the dicot model plant *Arabidopsis thaliana*. Here, we focus on the distribution and expression of the *PIN* and *PILS* gene families in areca palm (*Areca catechu*), a monocot tree. The whole genomic dataset of areca palm was used to identify twelve *AcPIN*s and eight *AcPILS*s, and a phylogenetic tree was constructed of *PINS* and *PILS* together with several other palm species, including the date palm (*Phoenix dactylifera*), oil palm (*Elaeis guineensis*), and coconut (*Cocos nucifera)*. We further analyzed the expression patterns of *AcPIN* and *AcPILS* in areca palm, and found that *AcPIN6* displayed an extremely high transcriptional abundance in the brace roots and was extremely stimulated in the lateral root primordium. This result implies that *AcPIN6* plays an important role in the growth and formation of brace roots, especially in lateral root initiation. We also overexpressed *AcPIN6* and *AcPIN6–eGFP* in *Arabidopsis*, and the results revealed that the PIN6 localized on the plasma membrane and affected auxin-related phenomena. Taken together, we analyzed the evolutionary relationships of *PINs* and *PILSs* in palm species, and the roles of *PIN6* in areca palm root formation. The results will improve the understanding of root system construction in large palm trees.

## 1. Introduction

Plants adapt to the environment by optimizing the size and structure of their vegetative organs, including roots, shoots, and leaves. The phytohormone auxin, the most abundant form of which is indole-3-acetic acid (IAA), acts as a key regulator in almost all aspects of plant growth and development. Polar auxin transport (PAT) mediated by auxin transporters distributes IAA from its synthesis cells to its effective loci, controlling cell division, elongation, and differentiation. According to the chemiosmotic hypothesis postulated in the 1970s, protonated IAA (IAAH) is predominant in the acidic extracellular spaces. The plasma membrane (PM) allows IAAH to freely diffuse into the cytoplasm, where the neutral environment deprotonates IAAH again, and IAAH becomes trapped inside the cytoplasm until it is actively transported by transmembrane-localized transporters. To date, four major families of auxin transporters have been discovered: PIN-FORMED (PIN), ATP-binding cassette family B (ABCB), AUXIN1/LIKE-AUX1s (AUX/LAX), and PIN-LIKES (PILS).

Among these families, members of the PIN-FORMED (PIN) family have been the most widely studied. In the model plant *Arabidopsis thaliana,* eight *PIN* genes (*AtPIN1–AtPIN8*) have been identified that differ in the length of the hydrophilic loop in the middle of their polypeptide chain [1]. Long PIN proteins, namely PIN1–4 and 7, are located at the PM, and determine the cell-to-cell PAT [2]. Two short PIN proteins, namely *AtPIN5* and *AtPIN8*, have a shorter central hydrophilic domain, and have been shown to localize in the endoplasmic reticulum (ER), suggesting that they may play a role in maintaining intracellular auxin homeostasis [3,4,5]. Notably, whether *AtPIN6* belongs to the classification of long or short PINs is controversial. Previous research found that *AtPIN6* was localized to both the PM and ER, and showed high sequence similarity with long *PINs.* The PILS transporter was identified based on its similarity to the PIN protein [6]. Seven members of the *PILS* gene family in *Arabidopsis* have been demonstrated to contribute to intracellular auxin transport, and transport auxin to the ER, attenuating the cellular response to auxin [6,7,8]. ABCB is subfamily B of the ATP binding cassette superfamily, which contains membrane transporters that can provide force by hydrolyzing ATP to transport chemicals and ions across the PM. *Arabidopsis* has 22 ABCBs. Among them, ABCB1/4/14/15/19/21 are involved in the cross-membrane transport of IAA [9]. AUX/LAX is encoded by a small gene family with four highly conserved protein-coding genes, including *AUX1* and *LAX1/2/3* [10]. These proteins act as auxin importers to transport IAA from intracellular spaces into cells. All of these auxin transporters together construct a complex network to guide the tissue-specific distribution of IAA, therefore optimizing the construction of plant organs.

Most of the current knowledge of PAT and auxin transporters has been obtained from *Arabidopsis*, a small, herbaceous, annual dicot plant. However, increasing data suggest that the PINs and PILS have crucial roles in the PAT process of monocots. For example, 12 *PIN* genes have been characterized in both *Oryza sativa* and *Zea mays* [11,12]. Six *PILS* genes have been identified in rice, and ten in maize [13]. PIN and PILS transporters have been reported to be related to auxin transport in these monocots. Monocots have quite different morphological structures compared to dicots. For example, unlike the tap root system of dicots, monocots have fibrous root systems composed of two kinds of adventitious roots: the crown roots and the brace roots. The crown roots initiate from subterranean nodes, while the brace roots initiate from the aerial stem nodes. The term “brace roots” is derived from the role of brace roots in lodging resistance, providing mechanical support to the plant body [14]. However, the mechanism of root system construction in monocots is still poorly studied.

The palm family (Arecaceae) is a widespread monocot family that comprises approximately 2500 species that inhabit almost all terrestrial environments in tropical and subtropical regions. To adapt to their environment, palms exhibit different body shapes, including shrubs, vines, herbs, and trees. Several economically important species, such as coconut, date palm, oil palm, and areca palm, are tall trees that may reach a height of more than 50 m [15]. Well-developed root systems are important for these trees to adapt to environmental challenges, including waterlogging, poorly developed soil profiles, and unstable soils.

This study focused on the areca palm (*Areca catechu* L.), one of the most widely cultured palms in East Africa, South Asia, and the Pacific islands [16]. Areca palm grows from a single main stem, has a crown of paripinnate leaves, bears simple evergreen leaves, and has an adventitious root system [17]. When areca seeds germinate, the germ then grows from the micropyle, and the adventitious roots grow from the coleoptile, forming the crown roots. After three to four years of growth, under the interstem epidermis near the surface of the ground, strong brace roots undergo organogenesis and extend into the ground, providing better mechanical support for the plant to reduce the effects of tropical rains and typhoons. This study identified twelve *AcPINs* and eight *AcPILSs* genes from the whole genome dataset of areca palm. Genetic and molecular biological approaches were used to reveal their evolutionary structures, spatial expression patterns, and functional roles in areca palm.

## 2. Results

### 2.1. Genome-Wide Identification of PIN and PILS Proteins in A. catechu

**Figure 1:**
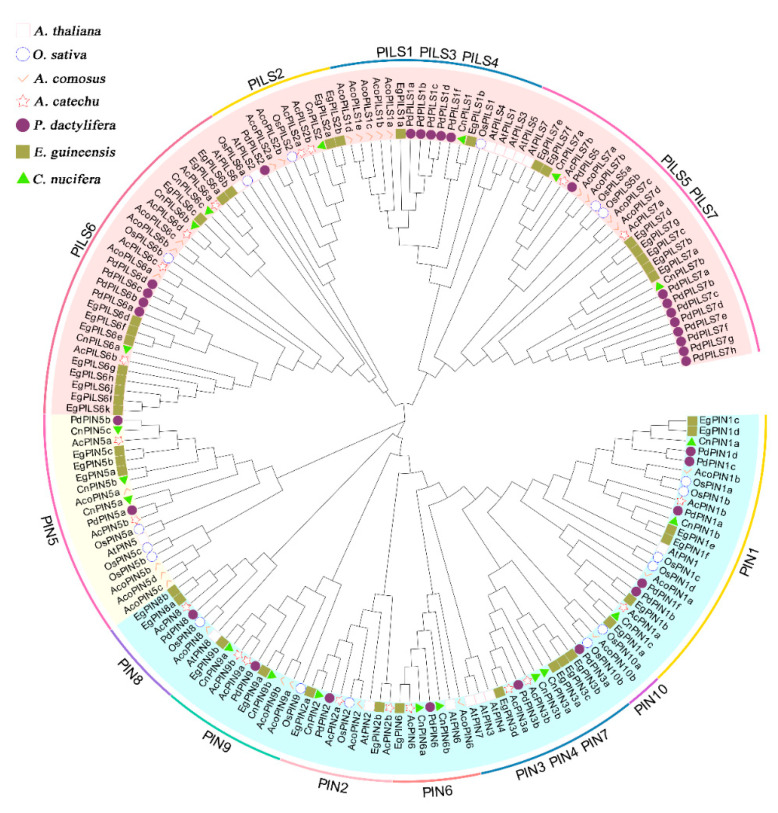
Phylogenetic analysis of PILS and PIN proteins from *A. catechu* (Ac), *Arabidopsis thaliana* (At), *Oryza sativa* (Os). *Ananas comosus* (Aco), *Cocos nucifera* (Cn), *Elaeis guineensis* (Eg), *Phoenix dactylifera* (Pd). Different PIN/PILS subfamilies are represented by different colors, and the different shapes represent different species.

Here, twelve *PIN* and eight *PILS* genes were identified from the whole genome sequence of *A. catechu* (*A. catechu* genomic data are available in the NCBI under the accession number: JAHSVC000000000). To understand the phylogenetic relationships of the *AcPIN*/*AcPILS* gene family with respect to *PIN/PILS* genes from different plant species, we constructed an evolutionary relationship tree based on the PIN/PILS protein sequences of *Arabidopsis*, *O. sativa*, *A. comosus*, *E. guineensis*, *C. nucifera*, and *P. dactylifera* (Figure 1). All AcPIN/AcPILS proteins were named according to their homologous relationships with known *Arabidopsis* and *O. sativa* PINs and PILSs. Phylogenetic analysis showed that the *A. catechu PIN* and *PILS* family genes were divided into 10 subfamilies, including *AcPIN1*, *AcPIN2*, *AcPIN3*, *AcPIN5*, *AcPIN6*, *AcPIN8*, *AcPIN9*, *AcPILS2*, *AcPILS6*, and *AcPILS7*. *AcPIN1*, *AcPIN2*, *AcPIN3*, *AcPIN5*, *AcPIN9*, *AcPILS2*, and *AcPILS7* had two copies, while *AcPILS6* had four copies (Figure 1). Among them, *PIN4* and *PIN7* were detected only in *Arabidopsis*. In monocots, *PIN3* was detected in all four palms with a different number of copies, but not in pineapple and rice (Figure 1 and Supplementary Materials, Table S1). These identified *AcPINs* and *AcPILSs* encoded proteins in *A. catechu* that ranged from 266 (*PIN5a*, *AC10G091050.1*) to 648 (*PIN3b*, *AC02G037360.1*) amino acids in length, with the pI value varying from 6.13 (*PILS7a*, *AC12G088170.1*) to 8.87 (*PILS6b*, *AC13G012690.1*) and molecular weights varying from 29.18 kD (*PIN5a*, *AC10G091050.1*) to 69.22 kD (*PIN3b*, *AC02G037360.1*) (Supplementary Materials, Table S2).

### 2.2. Chromosome Localization, Duplication, and Synteny of A. catechu PIN/PILS Genes

**Figure 2:**
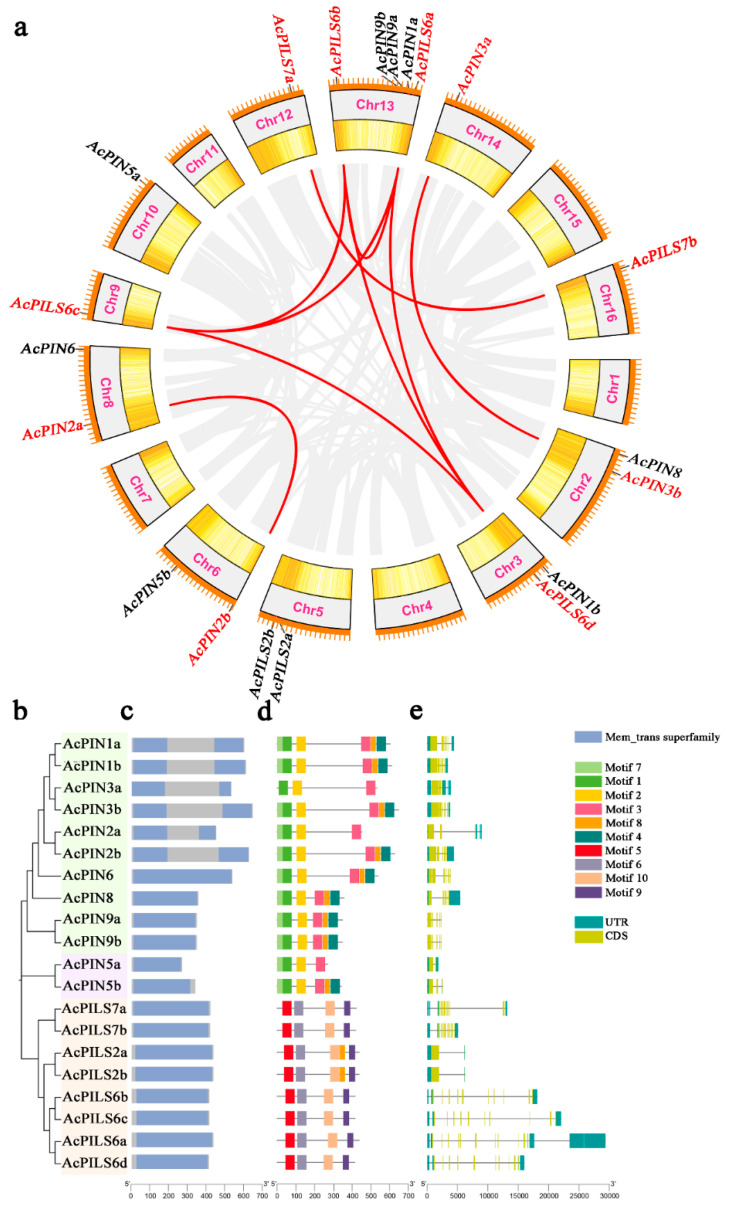
Synteny analysis and *AcPIN*/*AcPILS* gene structure and conserved motifs in *Areca catechu*. (**a**) Chromosomal locations and Synteny relationships of *AcPIN*/*AcPILS* genes. Colorful lines represent gene duplications. (**b**) Phylogenetic relationships. (**c**) Conserved protein domain. (**d**) Conserved motifs. The different colored boxes represent different motifs and their position in each AcPIN/AcPILS protein sequence. The scale bar indicates the number of amino acids. (**e**) Gene structure among *PIN* genes of *A. catechu*. The introns and exons are shown as black lines and yellow boxes, respectively. Atrovirens boxes represent the untranslated regions. The scale bar indicates number of nucleic acids (bp).

The twelve *AcPINs* and eight *AcPILSs* were randomly distributed across 11 of the 16 *A. catechu* chromosomes. Chromosomes 9, 10, 12, 14, and 16 each contained a single *AcPIN* or *AcPILS* gene; chromosomes 2, 3, 5, 6, and 8 each contained two *AcPIN* or *AcPILS* genes; chromosome 13 contained five *AcPIN* or *AcPILS* genes (Figure 2a). Importantly, it was found that most *AcPIN* and *AcPILS* genes were located at both ends of chromosomes (Figure 2a).

Gene duplication events usually include whole-genome duplication (WGD), tandem duplication, and segmental duplication. To investigate the diversification of duplication gene expansion patterns, this study identified five different categories of gene duplication events in *A. catechu*, including WGD events, tandem duplication events, proximal duplication events, transposed duplication events, and dispersed duplication events. Among them, fourteen *PIN/PILS* gene pairs were identified in *A. catechu* dispersed duplication events, five gene pairs in *A. catechu* transposed duplication events, and six gene pairs in *A. catechu* WGD events (Supplementary Materials, Table S3). To further investigate the syntenic relationships of *A. catechu PIN/PILS*, synteny analysis within family members was performed. Two pairs of syntenic *AcPIN* genes (*AcPIN2a*/*AcPIN2b* and *AcPIN3a*/*AcPIN3b*) and seven pairs of syntenic *AcPILS* genes (*AcPILS6a*/*AcPILS6c*, *AcPILS6a*/*AcPILS6d*, *AcPILS6b*/*AcPILS6a*, *AcPILS6b*/*AcPILS6c*, *AcPILS6b*/*AcPILS6d*, *AcPILS6d*/*AcPILS6c*, and *AcPILS7a*/*AcPILS7b*) were found in *A. catechu* (Figure 2a). In order to explore what type of selective pressure determined the divergence process of *AcPIN* and *AcPILS* after replication, the non-synonymous (Ka) and synonymous nucleotide substitutions (Ks) and their ratio (Ka/Ks) were calculated among the *AcPIN* and *AcPILS* gene pairs. The results showed all *AcPIN* and *AcPILS* paralog pairs had a ratio of Ka/Ks that was less than 1 (Supplementary Materials, Table S4).

### 2.3. Analysis of Conserved Motifs and Gene Structures of A. catechu PIN/PILS Genes

The *A. catechu* PIN/PILS proteins exhibited a highly conserved hydrophobicity profile, with two hydrophobic segments located at the N- and C-termini, being linked with a central hydrophilic loop (Supplementary Materials, Figure S1). All AcPIN and AcPILS proteins possessed six to ten transmembrane segments. AcPINs and AcPILSs could be classified as long and short PINs based on the length of the predicted protein and the central hydrophilic loop. The long PINs consisted of seven members, including all genes from the PIN1, PIN2, PIN3, and PIN6 groups; the short PINs and PILSs contained 13 members from the PIN5, PIN8, PIN9, and PILS groups (Supplementary Materials, Figure S1 and Table S1). Most “Long” PINs in A. catechu have two conserved phosphorylation sites (TPRXS motif), including AcPIN1a, AcPIN1b, AcPIN2a, AcPIN2a, AcPIN3a and AcPIN3b. “Short” PINs and AcPIN3b have no phosphorylation site, and AcPIN6 has only one site (Supplementary Materials, Figure S2).

Phylogenetic analysis showed that the long and short PIN proteins were evolutionarily different (Figure 2b). This study also identified the conserved domains and conserved motifs of AcPIN/AcPILS proteins (Figure 2c,d). Ten conserved protein motifs were discovered using MEME, and were characterized with high conservation in both their combination and relative position. All of the AcPILS members contained four motifs in a fixed order (motifs 5-6-10-9) at the C- and N-termini. However, AcPILS2 had more of motif 8 than the other six AcPILS members. Motif combinations of motif 7-1-2 were found in all AcPINs, except for AcPIN3a; at the N-terminal, AcPIN3a had only motif 2 and motif 1. Motifs 3-8-4 were found at the C-terminal in 75% of the AcPINs, and AcPIN2b, AcPIN3a, and AcPIN5a had only motif 3 (Figure 2d). In addition, the exons and introns of *AcPIN*/*AcPILS* genes were identified. The results showed that the number of introns ranged from 1 to 14, and *AcPILS6a* had most introns at 13. Most *PINs* contained five introns, while most *PILS*s contained 10 or 11 introns. Additionally, except for the *AcPILS2a* and *AcPILS2b* genes, most of the genes in the *AcPILS*s were longer than the *AcPIN*s (Figure 2e).

### 2.4. Expression Profile of AcPIN/AcPILS Genes in Various Organs and Tissues

**Figure 3:**
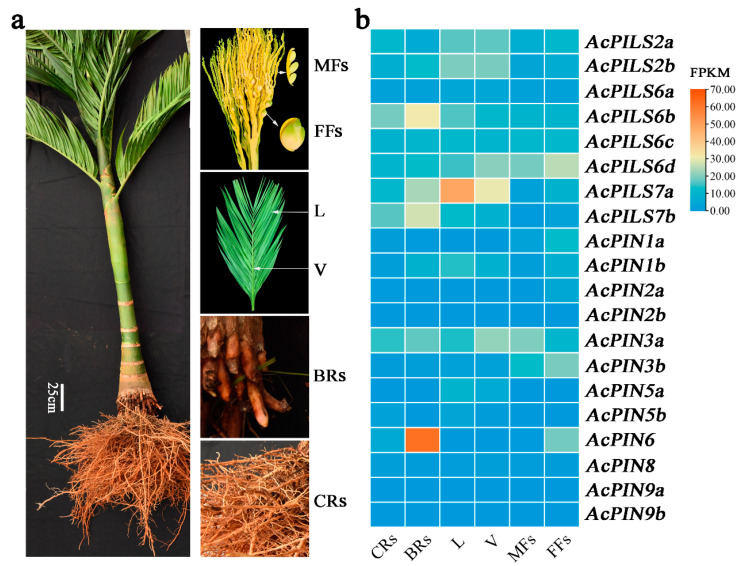
Morphological characteristics and *AcPIN* and *AcPILS* family gene expression profiles of *Areca catechu*. (**a**) Morphological characteristics *A. catechu*. CRs: Crown roots, BRs: Brace roots, L: Leaf, V: Vein, MFs: Male flowers, FFs: Female flowers. (**b**) Expression analysis of *AcPIN* and *AcPILS* genes of *A. catechu* showed that *AcPIN6* had the highest expression level in brace roots. Fragments per Kilobase Million (FPKM) values are visualized as a heat map.

To explore the expression patterns of *AcPIN*/*AcPILS* genes in different organs of *A. catechu*, this study analyzed the transcriptome data of six different organs and tissues (The RNA-seq data were downloaded in NCBI with accession number: PRJNA767949), namely the crown roots, brace roots, leaves, veins, male flowers, and female flowers (Figure 3a). The results showed that barely detectable expression or no expression was observed for *AcPIN2a*, *AcPIN2b*, *AcPIN5b*, *AcPIN8*, *AcPIN9a*, *AcPIN9b*, and *AcPILS6a* in all tested organs (Figure 3b). The *AcPIN3a*, *AcPILS6c*, and *AcPILS6d* expression levels remained relatively constant in all six organs. *AcPIN1b*, *AcPIN5a*, *AcPILS2a*, and *AcPILS2b* were mainly expressed in the leaves and veins. The expression of *AcPILS7a* was the highest in leaves and veins (Figure 3b). *AcPILS6b* and *AcPILS7a* were mainly expressed in crown roots and brace roots (Figure 3b). *AcPIN3a* and *AcPILS6d* were mainly expressed in male flowers, and *AcPIN3b*, *AcPIN6*, and *AcPILS7b* were mainly expressed in female flowers (Figure 3b). Notably, *AcPIN6* had the highest transcription abundance (FPKM > 60) in brace roots, a value that was significantly higher than that in crown roots (Figure 3b). The qRT-PCR showed that *AcPIN8* was expressed in male flowers. *AcPILS2a* and *AcPILS2b* showed the same expression level in all organs (Supplementary Materials, Figure S3). Overall, the results of qRT-PCR showed that the transcriptome data were reliable (Supplementary Materials, Figure S3).

### 2.5. Expression Dynamics of AcPIN6 in Brace Roots during Various Growth Stages and in Different Parts of Brace Roots

**Figure 4:**
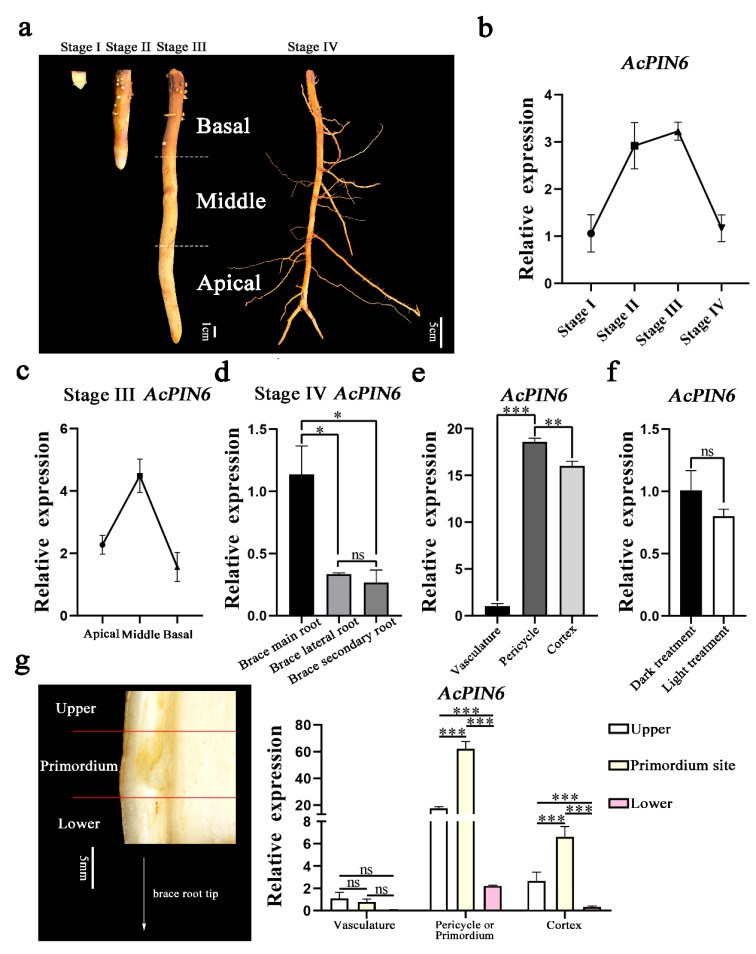
Expression analysis of *AcPIN6* in different parts and growth stages of the *A. catechu* brace root. (**a**) Brace root successive growth stages; A: the apical part of the brace root, M: the middle part of the brace root, B: the basal part of the brace root. (**b**) qRT-PCR analysis of *AcPIN6* during brace root growth. (**c**) qRT-PCR analysis of *AcPIN6* in different parts of the brace roots during Stage III. (**d**) qRT-PCR analysis of *AcPIN6* in the main brace root, brace lateral roots and brace secondary root during stage IV. (**e**) qRT-PCR analysis of *AcPIN6* in vasculature, pericycle and cortex. (**f**) qRT-PCR analysis of *AcPIN6* in brace roots pericycle under light and dark treatments. (**g**) Anatomical structure of the *A. catechu* brace root and qRT-PCR analysis of *AcPIN6* in different tissues after lateral root primordia. Error bars represent means ± SE from three independent experiments (ns: *p* > 0.05; * *p* < 0.05; ** *p* < 0.01; *** *p* < 0.001).

The transcriptome data and qRT-PCR results showed that the key PIN family gene, *AcPIN6*, had the highest expression level in the brace roots. To further observe the changes of *AcPIN6* in brace roots, this study established the dynamic expression level in brace roots during various growth stages. Four stages of root growth were selected, namely the beginning growth stage (Stage I), early growth stage (Stage II), middle growth stage (Stage III), and later growth stage (Stage IV) (Figure 4a). The *AcPIN6* expression was detected in brace roots during these four growth stages, and the results showed that the expression level of *AcPIN6* was low in the beginning growth stage, and gradually increased with the growth process. The expression level was highest in the middle growth stage, and gradually decreased in the late growth stage (Figure 4b). To further understand the expression of *AcPIN*6 in the same roots, middle growth stage brace roots were taken and divided into three parts: the apical part of the brace root (A), the middle part of the brace root (M), and the basal part of the brace root (B) (Figure 4a). The expression of *AcPIN6* in the middle section of brace roots was higher than that in the apical and basal parts of the brace roots (Figure 4c). *AcPIN6* was mainly expressed in the main brace root, with lower expression in the lateral and secondary roots (Figure 4d). Moreover, the expression level of *AcPIN6* in the pericycle was significantly higher than that in the cortex and vasculature (Figure 4e). In addition, this study treated both sides of the same position of the brace root with light and darkness, and found no significant difference in the expression of *AcPIN6* in the two positions of the pericycle (Figure 4f). After the occurrence of the lateral root primordium supporting the root, the expression level of *AcPIN6* in the lateral root primordium was significantly higher than that in the pericycle of the upper and lower parts (Figure 4g).

### 2.6. PIN6 Is Specifically Expressed in Brace Roots of Palms

**Figure 5:**
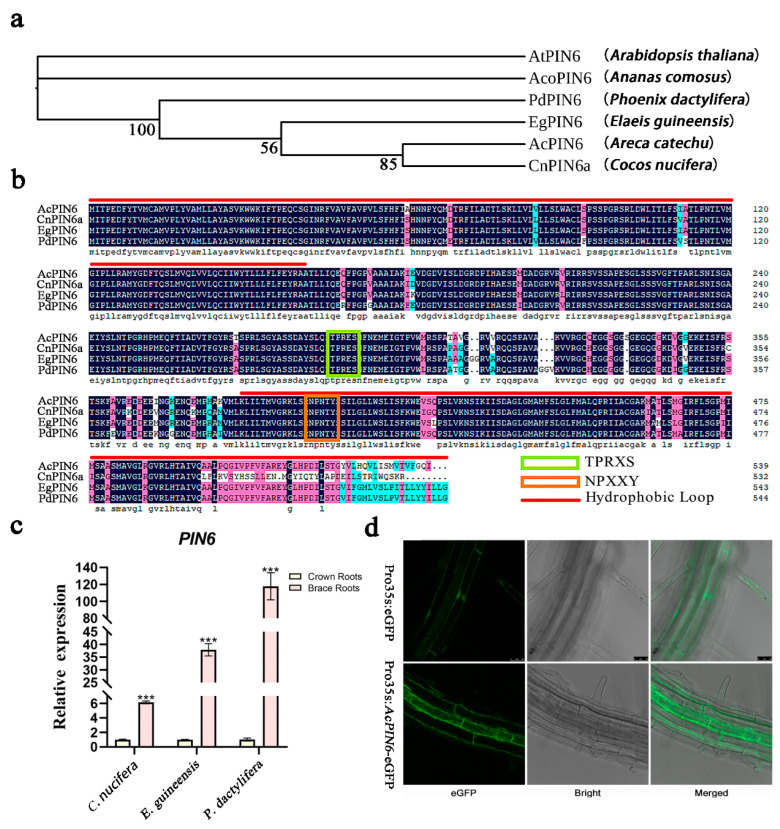
Palmae species PIN6 proteins amino acid sequence multiple alignment and expression levels in crown roots and brace roots. (**a**) Evolution of PIN6 in multiple species. (**b**) Amino acid sequence multiple alignment. The red line indicates a conserved transmembrane domain. The possible functional sites or elements are encircled by a box. (**c**) qRT-PCR analysis of *PIN6* in crown and brace roots of Palmae plants. (**d**) Subcellular localization of *AcPIN6*. Error bars represent mean ± SE from three independent experiments (*** *p* < 0.001).

Palm species grow in tropical environments, and many species have brace roots. To reveal whether *PIN6* plays a critical role in palm brace root development, an evolutionary analysis was conducted. The *PIN6* gene was examined in *Arabidopsis*, *A. comosus*, *E. guineensis*, *C. nucifera*, and *P. dactylifera*, and it was found that *A. catechu* had the closest evolutionary relationship with *C. nucifera* (Figure 5a). Sequence alignment showed that the PIN6 protein was highly conserved in Palmae, with a consistency of 93.52%. Furthermore, it was found that *PIN6* of all four species had a typical NPXXY domain near the C-terminus. Moreover, this study found a phosphorylation site associated with kinases in a hydrophilic loop, and there was a highly conserved TPRXS motif that could be phosphorylated by MPK4/6 kinase (Figure 5b). The expression levels of *PIN6* in the crown roots and brace roots of four Palmae plants were examined. The results showed that the expression level of *PIN6* in brace roots was significantly higher than that in crown roots in all four species (Figure 5c). In particular, the expression levels in the brace roots of *P. dactylifera* and *E. guineensis* were 120 times and 40 times higher than those in the crown roots, respectively (Figure 5c).

**Figure 6:**
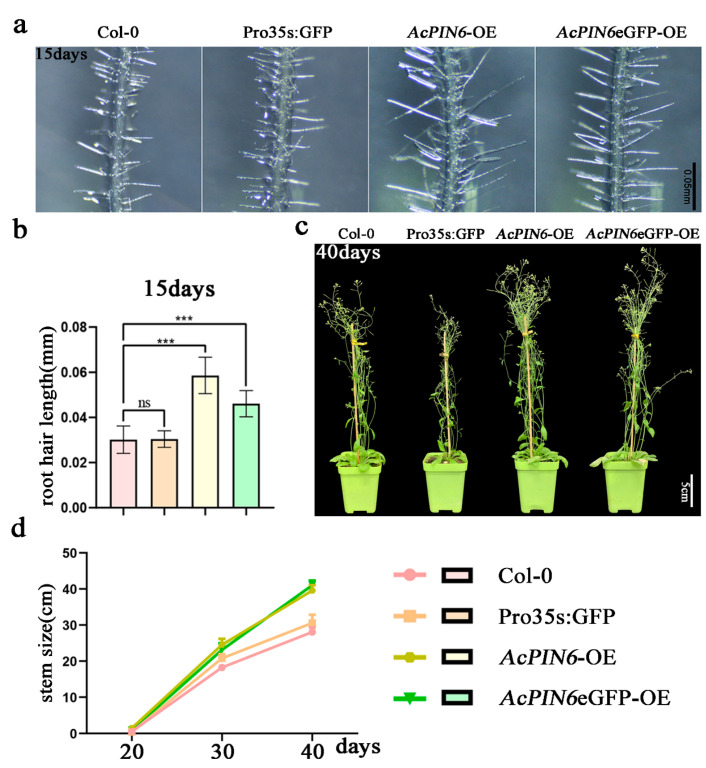
Phenotype of *Arabidopsis* overexpressing *AcPIN6.* (**a**) *AcPIN6*-overexpression root hair phenotype in *Arabidopsis* at 15 days. (**b**) Main root hair length (mm) at 15 days. (**c**) *AcPIN6*-overexpression phenotype in *Arabidopsis* grown for 40 days. (**d**) Plant height (cm) from 20–40 days. Error bars represent mean ± SE from three independent experiments (ns: *p* > 0.05; *** *p* < 0.001).

In order to further study the function of *PIN6* in the growth of brace roots, *AcPIN6* was fused into the C-terminal of eGFP to construct a pCAMBIA1300–*AcPIN6*–eGFP fusion expression vector. The resulting green fluorescence signal was mainly detected in the cell membrane, indicating that *AcPIN6* functioned in the cell membrane (Figure 5d). Furthermore, *AcPIN6*-overexpression *Arabidopsis* plants exhibited longer root hairs than Col-0 (Figure 6a,b), while no significant difference in root hair density was found between transgenic and Col-0 plants (Supplementary Materials, Figure S4c). After 20 days of incubation under the same growth conditions, compared with the Col-0 plants, the leaf length, width, and bolting rate of *AcPIN6*-overexpression lines were significantly increased (Supplementary Materials, Figures S4a and S3d–f). After 40 days of cultivation, there was no significant difference in leaf length between the *AcPIN6*-overexpression lines and the Col-0 plants, but the leaf width was still significantly higher than that of Col-0 (Figure 6c and Supplementary Materials, Figure S4g,h). In addition, compared with the Col-0 plants, the silique yield per plant, silique length, and plant height of the *AcPIN6*-overexpression lines were significantly increased (Figure 6c,d and Supplementary Materials, Figure S4j,k).

## 3. Discussion

Although auxin is the first discovered and most extensively studied phytohormone, almost all of the currently available knowledge about this phytohormone was obtained from a small model plants, including *Arabidopsis*, beans, rice and maize. Palms are widely distributed monocots that have quite different auxin-related phenotypes when compared to model plants. For example, areca palm lives in tropical regions and faces specific environmental challenges, such as strong wind stress and soil erosion in the rainy season. Therefore, this large tree developed a special structure to adapt to this environment. Its aboveground part has no branches nor lateral meristems, and its root system, with a combination of crown roots and brace roots, can support its height, allowing these palms to sometimes reach heights of 30–40 m.

The accurate distribution of IAA in roots is critical for the construction of root systems in plants [18]. IAA is synthesized in the stem cells of quick growth tissues, and then transported to the whole plant body to initiate or inhibit the construction of plant organs [19]. PIN and PILS are important auxin transport facilitators in this process. This study revealed the presence of twelve *PIN* and eight *PILS* genes in *A. catechu*, located on 11 separate chromosomes. A phylogenetic analysis was performed and the sequences of *AcPIN*s/*AcPILS*s were compared with those of six other species. The monocotyledon *PIN* family is usually enlarged by genome-wide duplication and the retention of multiple copies of similar proteins. The copy numbers of *PIN1*, *PIN3*, *PIN5*, *PIN9*, *PILS2*, *PILS6*, and *PILS7* genes in Palmae were increased compared to *Arabidopsis*. Previous studies showed that the *PIN3* and *PIN10* subfamilies were exclusive to dicots and monocots, respectively [12,20,21,22]. In this study, it was found that *PIN3* was retained and *PIN10* was lost in all four palm trees. In consideration of the functional redundancy of *PIN*s and *PILS*s, this study also found that the Ka/Ks ratios of all *AcPIN* and *AcPILS* paralog pairs were less than one, suggesting that these genes underwent purifying selection. Changes in the *PIN* and *PILS* gene numbers may not have been crucial events in the evolution of the special structure of areca palm trees.

Because tissue-specific expression patterns can provide evidence to understand the functional roles of genes, this study combined transcriptome and qRT-PCR to explore expression patterns in different organs and tissues. The expression profiles of *AcPIN* and *AcPILS* family genes were different in the six organs or tissues of *A. catechu*. A heat map was created to illustrate the expression abundances of *AcPINs* and *AcPILSs* in different plant organs, including crown roots, brace roots, leaves, leaf veins, and male and female flowers (Figure 3b). The heat map indicated an extremely high expression level of *AcPIN6* in the brace roots that merited further investigation. Therefore, this study explored its exact expression site using a series of qRT-PCR analyses. The results clearly indicated that *AcPIN6* had the highest expression level in the initiation site of lateral roots, namely, the primordium, located in the pericycle of developing brace roots. This finding was in agreement with the earlier conclusion that *PIN6* had a broad role in auxin-signaling-mediated developmental processes, such as lateral/adventitious root organogenesis, primary/lateral root development and growth, and root hair outgrowth [23,24,25]. This study then overexpressed *AcPIN6* and *AcPIN-eGFP* in the model plant *Arabidopsis*. The results indicated that AcPIN6-eGFP was localized to the cell membrane. The overexpression of *AcPIN6* resulted in several phenotypes, such as increased root hair length, plant height, and leaf length. In previous studies, *AtPIN6* overexpression caused a hairless phenotype in *Arabidopsis* [25,26]. Root hair growth is proportional to internal auxin levels in the root hair cell [27], and in *Arabidopsis*, overexpression of PIN6 might interfere with auxin availability in trichoblast and atrichoblast cells [23]. The *A. catechu* in this study is a tropical monocotyledon plant, and its roots do not have root hairs. We hypothesized that AcPIN6 has been subfunctional during the long-term evolution of *A. catechu* in the environment of tropical dry season and rainy season, therefore in *Arabidopsis*, it does not interfere with the auxins in trichoblast and atrichoblast cells. Taken together, it can be suggested that AcPIN6 is a membrane-localized protein related to auxin transport. However, the lack of reliable tools in genome modification and the relatively long growth period of palm trees prevent the collection of in vivo evidence from areca palm trees.

Because many palm tree species have similarly well-developed root systems, it can be further speculated that *PIN6* also plays an important role in the brace roots of other palm plants. This study tested coconut, date palm, and oil palm, for which the whole genomes have been published. It was found that *PIN6* was evolutionarily highly conserved in all four species. PIN protein non-conserved regions may also contain functional sites, which are often species-specific and related to species differences and sequence evolution [28,29,30]. qRT-PCR detection showed that the expression levels of *PIN6* in the brace roots of these three species were significantly higher than those in crown roots, suggesting that *PIN6* may play similar roles in the initiation of lateral roots in all four species investigated in this study.

In summary, this study identified twelve *PIN* and eight *PILS* genes from *A. catechu*. The *AcPIN*/*AcPILS* gene family was analyzed to investigate its evolutionary relationships, gene location on chromosomes, gene structure, and conserved motifs. It was found that the dicot-specific PIN3 was retained and the monocot-specific PIN10 was lost in the evolution process of all four palm trees studied. The expression profiles of the *AcPIN* and *AcPILS* family genes in the six organs or tissues examined showed that *AcPIN6* had a significantly high expression level in the brace roots. Accordingly, this study further analyzed the expression of *AcPIN6* in the brace roots and found that the expression of *AcPIN6* was highest in the lateral brace root primordia. The subcellular localization of overexpressed *AcPIN6-eGFP* in *Arabidopsis* showed that *AcPIN6* was localized on the cell membrane, and *Arabidopsis* plants overexpressing *AcPIN6* produced several phenotypes. Finally, it was found that the PIN6 protein was highly evolutionarily conserved in four palm trees, and the expression level of *PIN6* in the brace roots was significantly higher than that in the crown roots. These findings of the present study will provide useful information for further research on root system architecture in *A. catechu*.

## 4. Materials and Methods

### 4.1. Analysis of the PIN and PILS Gene Families

The whole-genome assembly of *A. catechu* protein sequences was downloaded from the NCBI database ((accessed on 1 December 2020) (JAHSVC000000000)). Functional annotation was filtered for the protein family database (Pfam) identifier of the PIN (PF03547) domain in *A. catechu*. All PIN and PILS protein sequences were extracted from the HMMER web server (http://hmmer.org/ (accessed on 15 December 2020)). Proteins with an E-value lower than 0.01 were retained, then the Pfam (http://pfam.sanger.ac.uk/search (accessed on 15 December 2020)), Conserved Domains Database (https://www.ncbi.nlm.nih.gov/Structure/cdd/cdd.shtml (accessed on 15 December 2020)), and SMART (http://smart.embl-heidelberg.de/ (accessed on 16 December 2020)) databases were used to confirm each predicted protein. The proteins meeting all of the above criteria were used for further study. The predicted molecular weights and the theoretical isoelectric points (pI) were determined using the ExPASy server (http://web.expasy.org/protparam/ (accessed on 1 May 2021)).

### 4.2. Phylogenetic Analysis of A. catechu PIN/PILS Gene Family

The PIN and PILS proteome sequences of *Arabidopsis*, *O. sativa*, *Ananas comosus*, *Elaeis guineensis*, *Cocos nucifera*, and *Phoenix dactylifera* were downloaded from Phytozome (https://phytozome.jgi.doe.gov (accessed on 10 March 2021)). Using the PIN and PILS protein alignment, a phylogenetic tree was inferred with MEGA 7.0 software (https://www.megasoftware.net/ (accessed on 20 March 2021)). Protein sequences were aligned with MUSCLE (https://www.ebi.ac.uk/Tools/msa/muscle/ (accessed on 20 March 2021)). The evolutionary relationships between proteins were inferred using the maximum likelihood method based on the JTT matrix-based model. The consistency of the phylogenetic estimate was evaluated with ultrafast bootstraps (1000 replicates each). This study suggests a consistent naming pattern for all *A. catechu PIN/PILS* genes, taking into account their phylogenetic relationships. Each gene name starts with an abbreviation for the species name, *A. catechu* (Ac), followed by the name of the most prominent *Arabidopsis* gene from this subfamily.

### 4.3. Chromosomal Distribution and Gene Synteny Analysis

Chromosome lengths and gene locations were obtained from the *A. catechu* genome annotation file, and TBtools software was used to visualize the gene locations on chromosomes [31]. *A. catechu* protein sequences were aligned to the protein sequences from *Arabidopsis*, *O. sativa*, *A. comosus*, *E. guineensis*, *C. nucifera*, and *P. dactylifera* using TBtools software. MCScanX was used with default parameters to identify gene duplication events and syntenic relationships, and the results were visualized using Circos and Dual Synteny Plot in TBtools. For Ka/Ks analysis, homologous gene pairs were identified with BLASTn using two criteria: (1) >75% sequence similarity and (2) an alignable region >75% of the length of the longer sequence. The Ka (non-synonymous substitution rate) and Ks (synonymous substitution rate) were calculated using TBtools. The transmembrane helices of PIN proteins were predicted using TMHMM2 (http://www.cbs.dtu.dk/services/TMHMM-2.0/ (accessed on 20 June 2021))

### 4.4. Transmembrane Topology, Conserved Motifs, and Gene Structure Analysis of AcPINs/AcPILSs

Protein transmembrane topology was predicted using the TMHHM server 2.0 (http://www.cbs. dtu.dk/services/TMHMM (accessed on 20 June 2021)). A conserved motif search was performed using the online tool Multi Em for Motif Elicitation (MEME Suite 4.12.0) (http://meme-suite.org/tools/meme (accessed on 25 June 2021)). MEME analysis parameters included a minimum width ≥ 6, a maximum width of 50, and a motif number of 10; all other parameters were set to default values. The exon/intron distributions of the *AcPINs/AcPILSs* structures were extracted from the general feature format (GFF3) file of *A. catechu* sequences and visualized using TBtools software.

### 4.5. Plant Materials and Treatment

The plant materials were taken from the experimental base of Haidian Campus of Hainan University, including *A. catechu E. guineensis*, *C. nucifera*, and *P. dactylifera.* The materials grow naturally in the environment, and the tree age is 4 years. Mixed samples of brace roots and crown roots of *E. guineensis*, *C. nucifera*, and *P. dactylifera* were taken at different periods. Using a clean blade and microscope, samples were taken at a low-temperature environment from the pericycle, cortex, and vasculature of the brace root of *A. catechu*. To investigate the possible function of light in response to expression of *AcPIN6* in the brace root pericycle, both sides of the *A. catechu* brace root were treated with dark and natural light for 10 h, respectively. All samples were frozen in liquid nitrogen, then stored at −80 °C for the following experiments. All experiments were repeated with three biological and technical replicates.

### 4.6. RNA Extraction, cDNA Synthesis and Quantitative Real-Time PCR Analysis

Expression analysis was conducted with total RNA isolated from various plant tissues (leaves, roots, and flowers) using an RNA extraction kit (Tiangen, Beijing, China, DP437). First-strand cDNA was generated using the TIANScript RT Kit (Tiangen, Beijing, China, KR104). The qRT-PCR was performed by a AceQ qPCR SYBR Green Master Mix (Vazyme, Nanjing, China, Q111-02). The primer pairs used for qRT-PCR were designed by Primer Premier 5.0 and β-actin was used as an internal control (Supplementary Materials, Table S5). The qRT-PCR program was as follows: 95 °C for 30 s, 40 cycles at 95 °C for 5 s, 60 °C for 30 s. The relative gene expressions were calculated using the 2^−ΔΔCT^method. There were three technical replicates for each biological replicate.

### 4.7. Transformation of Arabidopsis

The full-length CDS of the *AcPIN6* from *A. catechu* was cloned using a FastKing RT Kit (TIANGEN, Beijing, China) under the following PCR conditions: pre-denaturation of 10 min at 95 °C, amplification of 35 cycles at 95 °C for 30 s, 58 °C for 30 s and 72 °C for 1 min and a final extension of 10 min at 72 °C. The primer sequences were designed using SnapGene soft-ware and are listed in Supplementary Table S6. *Arabidopsis* (Ecotype Columbia-0) was used as a genetic background for both reporter gene lines, and Pro35S:*AcPIN6* and Pro35S:*AcPIN6-eGFP* constructs were generated by fusing full-length *AcPIN6* cDNA into a pCAMBIA1300 vector through restriction–ligation reactions. The resultant constructs were introduced in Columbia (Col-0) by *Agrobacterium*-mediated genetic transformation. Plants were grown on compost (Pindstrup Substrate, Guangzhou, Latvia). The growth conditions were as follows: 16 h light/8 h dark cycles, 300 μmol s^−1^ m^−2^ photosynthetically active radiation, and a temperature of 22 °C. For plates, seeds were sterilized with 10% NaClO and stratified at 4 °C for 2 days in the dark. Seedlings were grown vertically on 1/2 Murashige and Skoog (MS) medium (pH 5.7) with 1% (*w*/*v*) sucrose and 0.8% (*w*/*v*) agar.

### 4.8. Subcellular Localization

Three days after germination, the *Arabidopsis* seedlings were mounted in liquid ½ MS under a cover glass or under a small piece of growth medium agar in a chamber with a cover glass bottom. The meristematic root zone was imaged. Confocal images were obtained using a confocal laser scanning microscope (Leica TCS SP5 II; Leica Microsystems, Wetzlar, Germany) with a ×63 water immersion objective. The following light ranges were used: green fluorescent protein (GFP) (Ex 488 nm, Em 500–543 nm); 4′,6-diamidino-2-phenylindole (Ex 405 nm, Em 430–550 nm); chlorophyll autofluorescence (Ex 488 nm, Em 673–725 nm). The resulting images were adjusted for brightness and contrast. The picture processing was done by ZEN software (Carl Zeiss GmbH, Jena, Germany) and Photoshop CS6 (Adobe, San Jose, CA, USA).

### 4.9. Statistical Analysis

All statistical analyses were performed using GraphPad software (v.8.2). The data were analyzed using analysis of variance and presented as the mean ± SD (standard deviation), taking ns: *p* > 0.05; * *p* < 0.05; ** *p* < 0.01; *** *p* < 0.001 as levels of significance.

## Data Availability

The datasets presented in this study can be found in online repositories. The names of the repository/repositories and accession number(s) can be found in the article/Supplementary Material. The *A. catechu* genomic data is available in the National Center for Biotechnology Information (NCBI) under the accession number: JAHSVC000000000. The RNA-seq data were downloaded in NCBI with accession number: PRJNA767949.

## Supplementary Materials

The following supporting information can be downloaded at: https://www.mdpi.com/article/10.3390/plants12010033/s1, Figure S1: Predicted topology PIN/PILS proteins. These predictions were made by HMMTOP 2.0; Figure S2: *AcPIN* genes amino acid sequence multiple alignment; Figure S3: The *AcPIN/AcPILS gene*s expression pattern in different *A. catechu* organs for qRT-PCR. Figure S4: Phenotype of *Arabidopsis* overexpressing *AcPIN6*; Table S1: *PIN*/*PILS* family genes in *A. catechu* L. Table S2: Size of *PIN*/*PILS* family genes in different plant species. Table S3: Identification of *AcPIN*/*AcPILS* family gene duplication events. Table S4: KA and KS calculations of the *AcPIN*/*AcPILS* gene pairs. Table S5: The primers used for quantitative real-time PCR. Table S6: The primers used for Full-Length *AcPIN6* and *AcPIN6*-eGFP.

## References

[1] Peer W.A., Blakeslee J.J., Yang H., Murphy A.S. (2011). Seven things we think we know about auxin transport. Mol. Plant..

[2] Zhou J.J., Luo J. (2018). The PIN-FORMED auxin efflux carriers in plants. Int. J. Mol. Sci..

[3] Mravec J., Skůpa P., Bailly A., Hoyerová K., Krecek P., Bielach A., Petrásek J., Zhang J., Gaykova V., Stierhof Y.D. (2009). Subcellular homeostasis of phytohormone auxin is mediated by the ER localized PIN5 transporter. Nature.

[4] Ding Z., Wang B., Moreno I., Dupláková N., Simon S., Carraro N., Reemmer J., Pěnčík A., Chen X., Tejos R. (2012). ER-localized auxin transporter PIN8 regulates auxin homeostasis and male gametophyte development in Arabidopsis. Nat. Commun..

[5] Bennett T., Brockington S.F., Rothfels C., Graham S.W., Stevenson D., Kutchan T., Rolf M., Thomas P., Wong G.K., Leyser O. (2014). Paralogous radiations of PIN proteins with multiple origins of noncanonical PIN structure. Mol. Biol. Evol..

[6] Barbez E., Kubeš M., Rolčík J., Béziat C., Pěnčík A., Wang B., Rosquete M.R., Zhu J., Dobrev P.I., Lee Y. (2012). A novel putative auxin carrier family regulates intracellular auxin homeostasis in plants. Nature.

[7] Béziat C., Barbez E., Feraru M.I., Lucyshyn D., Kleine-Vehn J. (2017). Light triggers PILS-dependent reduction in nuclear auxin signalling for growth transition. Nat. Plants.

[8] Sauer M., Kleine-Vehn J. (2019). PIN-FORMED and PIN-LIKES auxin transport facilitators. Development.

[9] Cho M., Cho H.T. (2013). The function of ABCB transporters in auxin transport. Plant Signal Behav..

[10] Péret B., Swarup K., Ferguson A., Seth M., Yang Y., Dhondt S., James N., Casimiro I., Perry P., Syed A. (2012). AUX/LAX genes encode a family of auxin influx transporters that perform distinct functions during Arabidopsis development. Plant Cell.

[11] Miyashita Y., Takasugi T., Ito Y. (2010). Identification and expression analysis of *PIN* genes in rice. Plant Sci..

[12] Forestan C., Farinati S., Varotto S. (2012). The maize *PIN* gene family of auxin transporters. Front. Plant Sci..

[13] Feraru E., Vosolsobě S., Feraru M.I., Petrášek J., Kleine-Vehn J. (2012). Evolution and structural diversification of PILS putative auxin carriers in plants. Front. Plant Sci..

[14] Frank H., Woong J.P., Michaela S., Katrin W. (2004). From weeds to crops: Genetic analysis of root development in cereals. Trends Plant Sci..

[15] Hunter I.R., Bystriakova N. (2004). Encyclopedia of Forest Sciences.

[16] Srimany A., George C., Naik H.R., Pinto D.G., Chandrakumar N., Pradeep T. (2016). Development patterning and segregation of alkaloids in arece nut (seed of *Areca catechu*) revealed by magnetic resonance and mass spectrometry imaging. Phytochemistry.

[17] Peter K.V., Kurian A., Chopra V.L. (2003). Encyclopedia of Applied Plant Sciences.

[18] Saini S., Sharma I., Kaur N., Pati P.K. (2013). Auxin: A master regulator in plant root development. Plant Cell Rep..

[19] Marhava P., Bassukas A.E.L., Zourelidou M., Kolb M., Moret B., Fastner A., Schulze W.X., Cattaneo P., Hammes U.Z., Schwechheimer C. (2018). A molecular rheostat adjusts auxin flux to promote root protophloem differentiation. Nature.

[20] Balzan S., Johal G.S., Carraro N. (2014). The role of auxin transporters in monocots development. Front Plant Sci..

[21] Adamowski M., Friml J. (2015). PIN-dependent auxin transport: Action, regulation, and evolution. Plant Cell.

[22] Wang Y.Q., Chai C.L., Valliyodan B., Maupin C., Annen B., Nguyen H.T. (2015). Genome-wide analysis and expression profiling of the *PIN* auxin transporter gene family in soybean (*Glycine max*). BMC Genom..

[23] Cazzonelli C.I., Vanstraelen M., Simon S., Yin K., Carron-Arthur A., Nisar N., Tarle G., Cuttriss A.J., Searle I.R., Benkova E. (2013). Role of the Arabidopsis PIN6 auxin transporter in auxin homeostasis and auxin-mediated development. PLoS ONE.

[24] Nisar N., Cuttriss A.J., Pogson B.J., Cazzonelli C.I. (2014). The promoter of the *Arabidopsis PIN6* auxin transporter enabled strong expression in the vasculature of roots, leaves, floral stems and reproductive organs. Plant Signal Behav..

[25] Simon S., Skůpa P., Viaene T., Zwiewka M., Tejos R., Klíma P., Čarná M., Rolčík J., De Rycke R., Moreno I. (2016). PIN6 auxin transporter at endoplasmic reticulum and plasma membrane mediates auxin homeostasis and organogenesis in Arabidopsis. New Phytol..

[26] Ditengou F.A., Gomes D., Nziengui H., Kochersperger P., Lasok H., Medeiros V., Paponov I.A., Nagy S.K., Nádai T.V., Mészáros T. (2017). Characterization of auxin transporter PIN6 plasma membrane targeting reveals a function for *PIN6* in plant bolting. New Phytol..

[27] Ganguly A., Lee S.H., Cho M., Lee O.R., Yoo H., Cho H.T. (2010). Differential auxin-transporting activities of PIN-FORMED proteins in *Arabidopsis* root hair cells. Plant Physiol..

[28] Barbosa I.C.R., Schwechheimer C. (2014). Dynamic control of auxin transport-dependent growth by AGCVIII protein kinases. Curr. Opin. Plant Biol..

[29] Weller B., Zourelidou M., Frank L., Barbosa I.C., Fastner A., Richter S., Jürgens G., Hammes U.Z., Schwechheimer C. (2017). Dynamic PIN-FORMED auxin efflux carrier phosphorylation at the plasma membrane controls auxin efflux-dependent growth. Proc. Natl. Acad. Sci. USA.

[30] Zwiewka M., Bilanovičová V., Seifu Y.W., Nodzyński T. (2019). The nuts and bolts of PIN auxin efflux carriers. Front. Plant Sci..

[31] Chen C., Chen H., Zhang Y., Thomas H.R., Frank M.H., He Y., Xia R. (2020). TBtools: An integrative toolkit developed for interactive analyses of big biological data. Mol. Plant.

