# Supplementary material for: Genome-Wide Identification of PIN and PILS Gene Families in Areca catechu and the Potential Role of AcPIN6 in Lateral Brace Root Formation
Source: Plants (Basel). 2022 Dec 21;12(1):33. doi: 10.3390/plants12010033 (PMC9824567; doi:10.3390/plants12010033)
Supplement: Supplementary file 1 [file plants-12-00033-s001.zip › Supplementary figure.pdf]

TMHMM posterior probabilities for *AcPINs/AcPILSs*

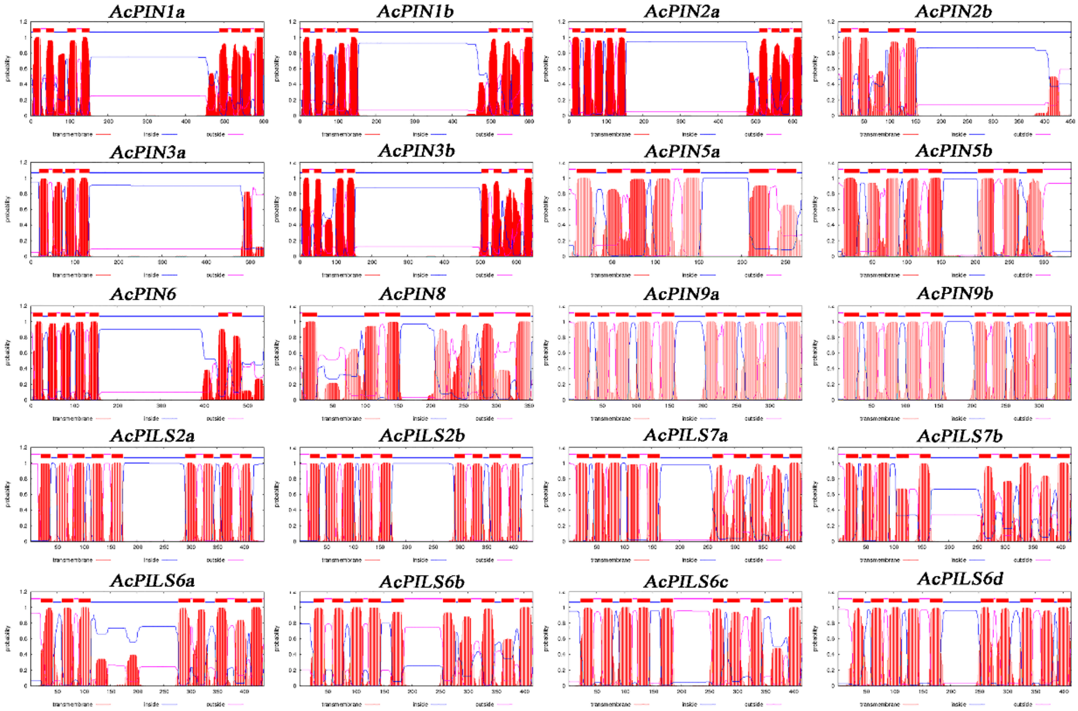

Figure S1. Predicted topology PIN/PILS proteins. These predictions were made by HMMTOP 2.0.

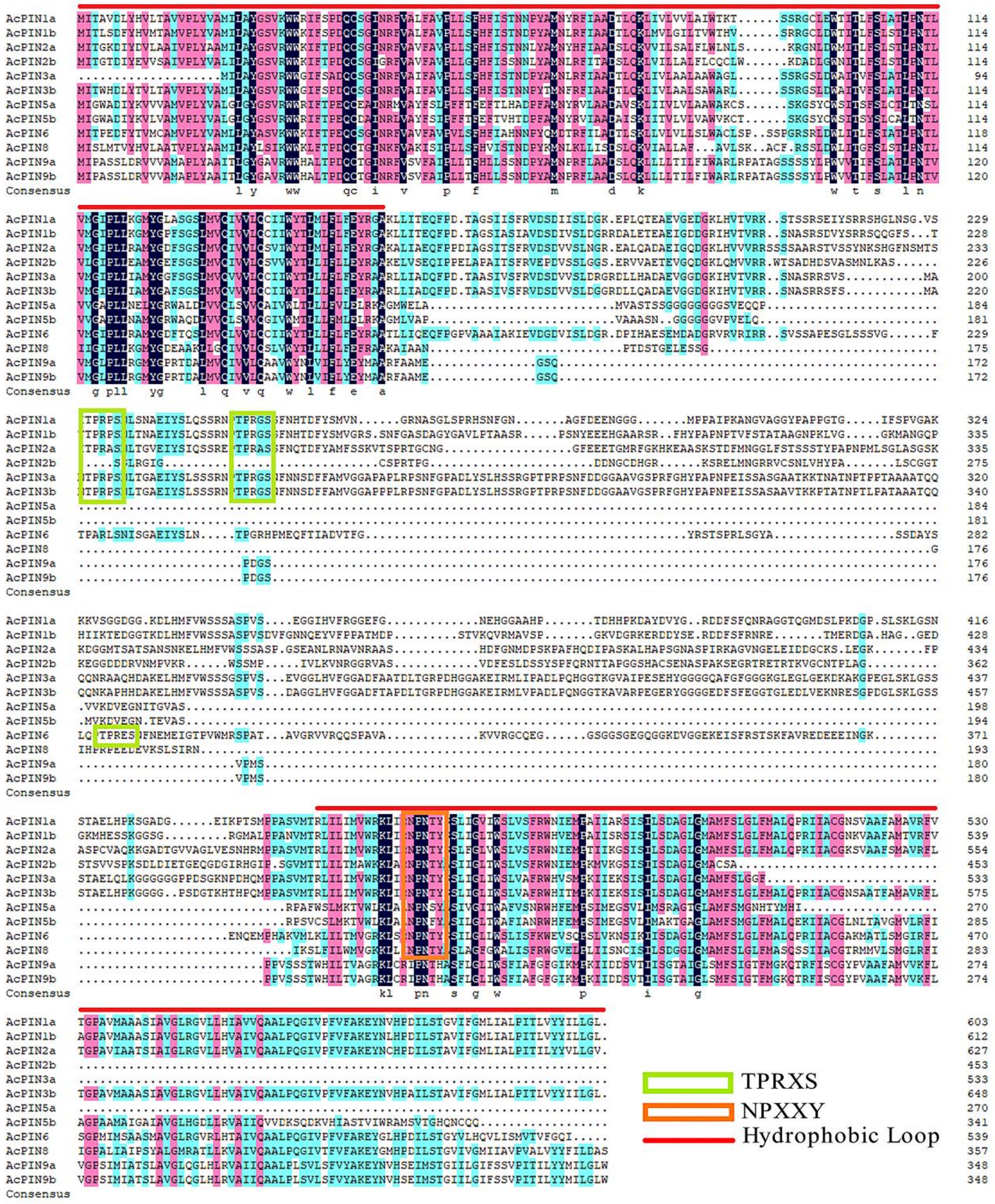

Figure S2. AcPIN genes amino acid sequence multiple alignment. The red line indicates a conserved transmembrane domain. The possible functional sites or elements are encircled by a box.

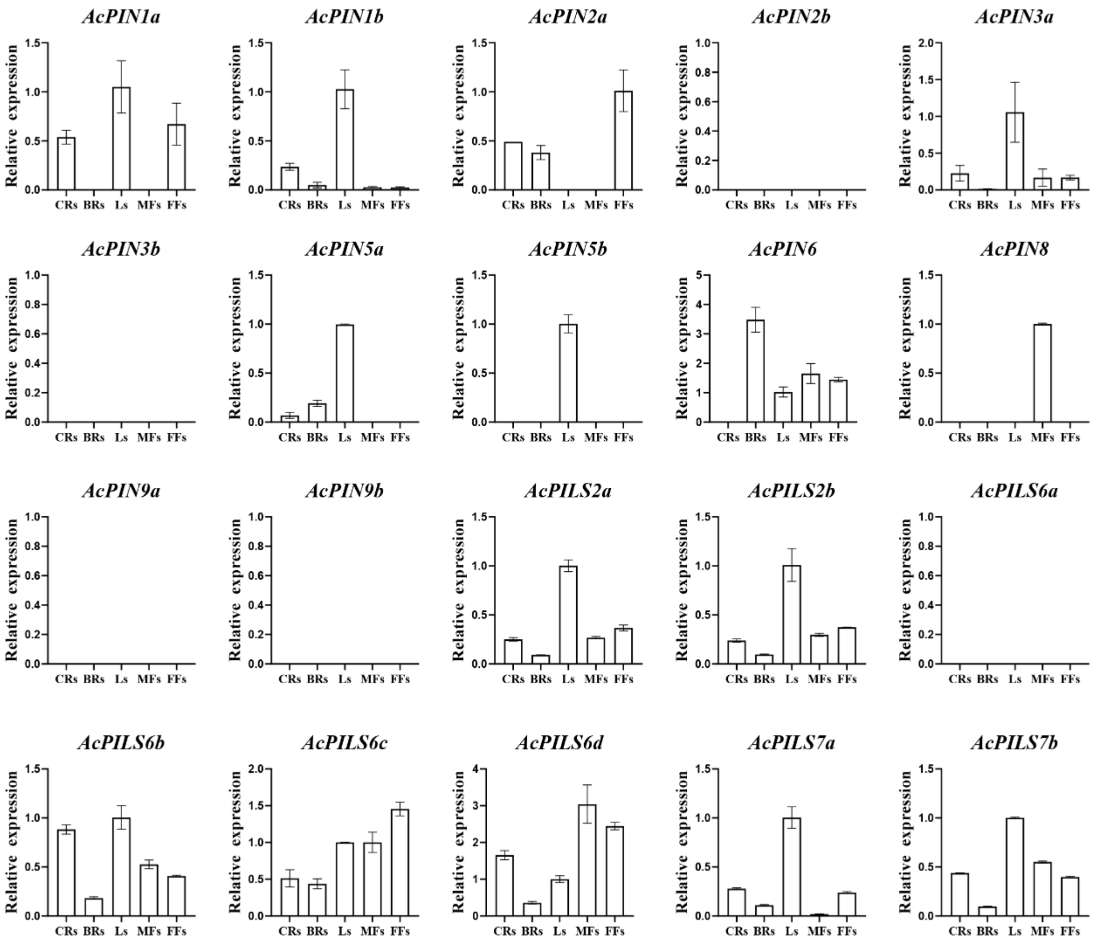

Figure S3. The *AcPIN*/*AcPILS* genes expression pattern in different *A. catechu* organs for qRT-PCR. qRT-PCR experiments were performed on three biological replicates. Error bars represent means ±SE from three independent experiments.

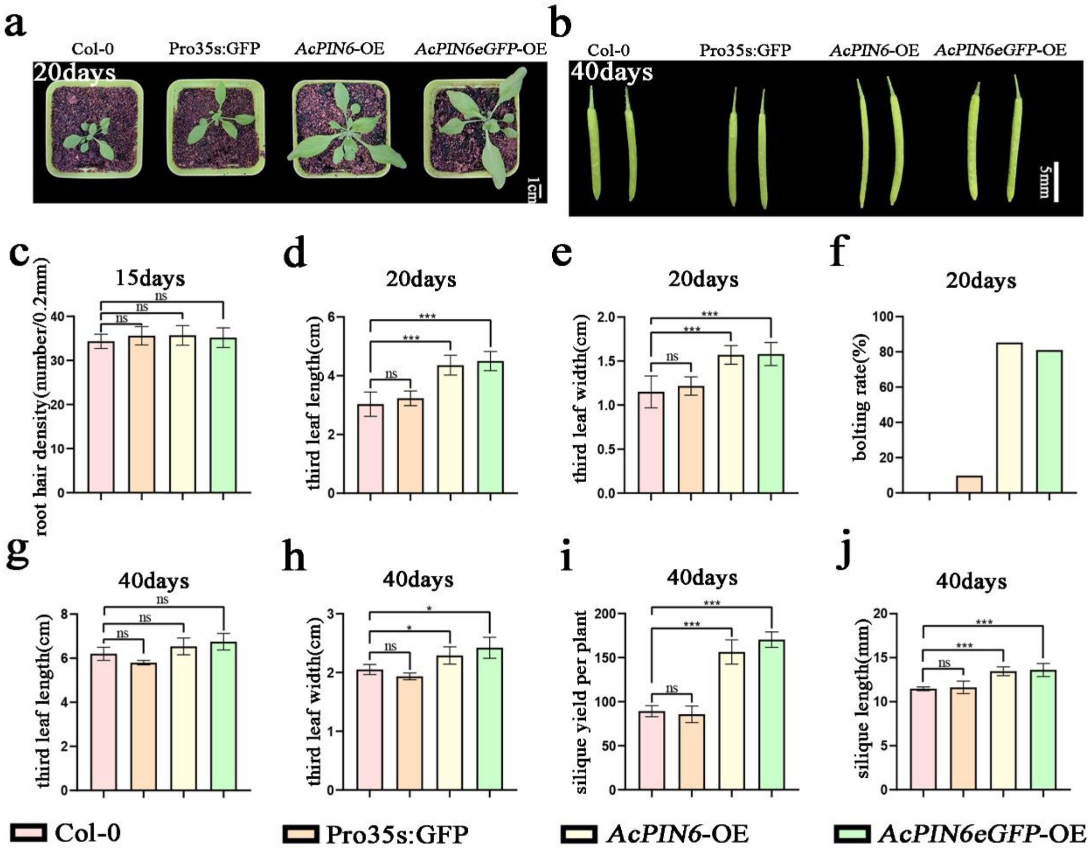

Figure S4. Phenotype of *Arabidopsis* overexpressing *AcPIN6.* (a). *AcPIN6* overexpression phenotype in *Arabidopsis* grows for 20 days. (b). *AcPIN6* overexpressed a 40-day silique phenotype in *Arabidopsis.* (c). root hair density(number/0.2mm) in 15 days. (d-f). leaf length, leaf width and bolting rate in 20 days. (g-j). leaf length(cm), leaf width(cm), silique yield per plant and silique length(mm) in 40 days. Error bars represent means ±SE from three independent experiments (ns: $p > 0.05$; *$p < 0.05$; **$p < 0.01$; ***$p < 0.001$).
